# Dental Bill of the State of New Jersey

**Published:** 1884-04

**Authors:** 


### DENTAL BILL OF THE STATE OF NEW JERSEY.

The following bill has passed both branches of the Legislature of the State of New Jersey for a copy of which we are indebted to Dr. Jas. G. Palmer, of New Brunswick.

"A supplement to an act entitled 'An Act to Regulate the Practice of Dentistry, and to protect the people against empiricism in relation thereto, in the State of New Jersey,' approved March fourteenth, one thousand eight hundred and seventy-three."

*Be it enacted* by the Senate and General Assembly of the State of New Jersey, that the first section of the act to which this is a supplement shall be amended so as to read as follows:

*Be it enacted*, by the Senate and General Assembly of the State of New Jersey, that from and after the passage of this act it shall be unlawful for any person not now lawfully practicing to engage in the practice of dentistry in the State of New Jersey unless said person has graduated and received a diploma from the faculty of a reputable dental college chartered under the authority of some one of the United States, and that any person hereafter engaging in the practice of dentistry in the State shall within one month after commencing such practice register his name in a book, kept for that purpose in the county clerk's office of the county in which he shall have engaged in the practice of dentistry giving his name and name of the dental college of which he is a graduate, and the name of the place in which he shall have engaged in practice, and for which registry the said county clerk shall be entitled to demand and receive from each person registering the sum of fifty cents, and any person violating any ot the provisions of this act shall be liable to the penalties prescribed in the sixth section of the act to which this is a supplement."

